# Ten-Year Experience in the Treatment of Cancers of Unknown Primary from Poland: From Overall Survival to Factors Influencing Response in the Prepersonalization Era

**DOI:** 10.3390/cancers18040565

**Published:** 2026-02-09

**Authors:** Konrad Tałasiewicz, Aleksandra Czachowska, Marcin Folwarski, Iwona Ługowska, Dominika Jaxa-Larecka, Aleksandra Kapała

**Affiliations:** 1 Department of Oncology Diagnostics and Palliative Medicine, Maria Sklodowska-Curie National Research Institute of Oncology in Warsaw (MSCI), Roentgena 5, 02-781 Warsaw, Poland; 2 Department of Clinical Nutrition and Dietetics, Medical University of Gdańsk, Dębinki 7, 80-211 Gdańsk, Poland; 3 Nicolaus Copernicus Hospital, Nowe Ogrody 1-6, 80-803 Gdańsk, Poland; 4 Center of Excellence in Precision Oncology, Maria Sklodowska-Curie National Research Institute of Oncology in Warsaw (MSCI), Roentgena 5, 02-781 Warsaw, Poland; 5 Department of Clinical Nutrition, Maria Sklodowska-Curie National Research Institute of Oncology in Warsaw (MSCI), Roentgena 5, 02-781 Warsaw, Poland

**Keywords:** cancer of unknown primary origin, primary cancer of unknown origin, prognosis, survival, NLR

## Abstract

**Background:** Cancer of unknown primary represents 3–5% of all cancer cases. This study aimed to identify the clinical and pathological factors affecting survival in these patients before the introduction of next-generation sequencing in modern molecular diagnostics. **Methods:** A retrospective cross-sectional observational study involving 149 patients with CUP at the Maria Sklodowska-Curie National Research Institute of Oncology in Warsaw, Poland, was conducted between 2006 and 2016. Clinical records were analyzed to gather demographic, clinical, and pathological data. Statistical analyses, including the Kaplan—Meier method, estimated overall survival (OS), and Cox proportional hazards modeling, were used to identify significant clinical factors associated with survival. **Results:** The mean age was 59 years, and 57% of the patients were male. Most patients (59.44%) had an ECOG of 0–1. The most common metastatic sites were the lymph nodes (63.64%), liver (41.26%), bones (23.78) and lungs (21.68%). The median overall survival for the cohort was 7.1 months, with notable survival disparities based on several clinical and laboratory factors. Multivariate analysis revealed that ECOG score >1 (HR = 1.69, 95% CI: 1.10–2.60, *p* = 0.016), elevated ALP (HR = 1.81, 95% CI: 1.20–2.74, *p* = 0.005), elevated calcium level (HR = 2.08, 95% CI: 1.05–4.13, *p* = 0.037), and NLR ≥2.61 (HR = 2.55, 95% CI: 1.67–3.89, *p* < 0.001) were independent predictors of poorer survival. Elevated LDH showed a consistent trend toward poorer survival but did not reach significance in the fully adjusted model (HR = 1.50, 95% CI: 0.99–2.27, *p* = 0.054). **Conclusions:** This study provides insights into the survival and prognostic factors influencing CUP patients, assisting clinicians in treatment eligibility assessments and guiding future investigations.

## Simple Summary

Cancer of unknown primary is a rare and challenging condition in which the cancer has already spread, but its original site cannot be identified. Despite advances in cancer diagnostics, the survival of these patients has remained poor for many years. In this study, we analyzed real-world clinical data from patients treated before modern molecular testing was widely available, in order to identify simple clinical and laboratory factors that influence survival at the time of diagnosis. We found that general health status, routine blood test results, and markers of inflammation were strongly associated with patient outcomes. These findings highlight that widely available clinical information can help estimate prognosis and support treatment decisions early in the disease course. Our results provide a useful reference point for clinicians and researchers and may serve as a baseline for comparison with outcomes in the current era of molecularly guided therapies.

## 1. Introduction

Cancer of unknown primary (CUP) encompasses a wide range of oncological diagnoses, accounting for approximately 3–5% of all cancer cases in historical data. Recently published studies indicate a decline in the incidence of this condition [1]. According to the Polish National Cancer Registry (2023), patients diagnosed with the ICD-10 codes C76-C80 represent less than 2% of overall cancer morbidity [2].

While the decline in incidence may be attributed to advancements in diagnostic techniques, treatment outcomes have unfortunately remained unchanged over the past 30 years [3]. After a mandatory clinical assessment that includes patient history, physical examination, blood tests, CT scans, and mammography for women, patients are classified into two categories on the basis of clinical and pathological features: favorable site-specific CUP, which has a better prognosis than cancers with known primary sites, and unfavorable CUP, which presents a poor prognosis with an expected survival of less than one year [4]. Unfavorable CUP patients pose significant challenges, making up the majority of CUPs (up to 80%) [4].

Although numerous clinical factors influence prognosis, the most commonly utilized factors in practice are performance status and lactate dehydrogenase (LDH) levels at diagnosis. Specifically, patients with good prognostic indicators have an Eastern Cooperative Oncology Group (ECOG) performance status of 0 or 1 and normal LDH levels, whereas those with poor prognostic indicators have an ECOG performance status greater than 1 or elevated LDH levels [5].

Currently, the standard treatment for unfavorable CUP is cytotoxic chemotherapy. Platinum-based doublets are the standard of care and are supported primarily by randomized phase II trials and meta-analyses, although they have not been validated in adequately planned randomized phase III trials [6,7,8]. The evidence for immunotherapy and personalized treatment approaches is based on agnostic strategies or several small phase I/II trials, with reimbursement being limited owing to unclear benefits [9,10]. In this era of next-generation sequencing (NGS), there is increasing evidence that mutational profiles can aid in treatment planning, serve as prognostic and predictive markers, help identify the tissue of origin, and reveal targetable alterations.

In our study, we analyzed real-world evidence (RWE) data, including clinical and pathological information, from the decade prior to the widespread use of next-generation sequencing and advanced molecular techniques in diagnostics. The primary goal of the study was to identify the factors that influence survival. Secondary objectives included the characterization of treatment patterns and outcomes in a real-world cohort of CUP patients and the exploratory evaluation of routinely available laboratory parameters in the pre-next-generation sequencing era. Currently, combining knowledge of clinical factors with the results of molecular analyses can support more effective clinical decision-making.

## 2. Materials and Methods

### 2.1. Study Design and Study Group Characteristics

In a cross-sectional observational study, we retrospectively analyzed the medical records of patients with a diagnosis of cancer of unknown primary treated at the Maria Sklodowska-Curie National Research Institute of Oncology in Warsaw, Poland, between January 2006 and December 2016.

Eligible persons were adult patients diagnosed with cancer of unknown primary (ICD-10 codes C76-C80). Inclusion criteria were availability of retrospective medical records, at least cytopathology diagnosis of carcinoma, completion of standard diagnostic workup including clinical examination and whole-body computed tomography, and sufficient clinical and pathological data for survival analysis. Exclusion criteria included insufficient clinical or pathological data and identification or strong suspicion of a primary tumor site during diagnostic evaluation.

A total of 149 patients were initially identified. After applying the inclusion and exclusion criteria, 143 patients were included in the final analysis; six patients were excluded because of insufficient data or suspected identification of the primary tumor site.

All included patients had at least a cytopathological diagnosis of carcinoma and underwent clinical examinations as well as whole-body computed tomography (CT). Additional diagnostic methods, in accordance with best clinical practices, were performed according to local standards and existing guidelines at the time of diagnosis.

None of the study subjects underwent next-generation sequencing or tests for molecular alterations, as these methods were not standard at our institution during the study period.

### 2.2. Parameters Evaluated

Data collected included age, sex, tumor histology (including diagnosis date and pathology results), number of metastatic sites, performance status assessed by the Eastern Cooperative Oncology Group (ECOG), treatment type, and laboratory test results before and during treatment: complete blood count (CBC), calcium (Ca^2+^), lactate dehydrogenase (LDH), alkaline phosphatase (ALP), neutrophil-lymphocyte ratio (NLR), and platelet-lymphocyte ratio (PLR). For each patient, a single set of pretreatment values was analyzed, corresponding to the time of CUP diagnosis and before qualification for systemic therapy or other oncological treatment. Although some patients underwent multiple hospitalizations during the study period, each patient contributed only one set of baseline clinical and laboratory data obtained during the diagnostic workup prior to treatment initiation. The number of metastatic sites was based on the organs involved, with the NLR and PLR calculated as simple ratios by dividing neutrophil and platelet counts by lymphocyte counts in the peripheral blood. CUP cases were stratified into two prognostic risk groups: favorable (ECOG 0–1 and normal LDH) and unfavorable (ECOG 2–4 and/or elevated LDH), based on established prognostic factors recommended for clinical risk assessment in patients with cancer of unknown primary [11]. Prognostic risk groups were constructed retrospectively for the purposes of statistical analysis, using ECOG and LDH levels recorded at the time of CUP diagnosis. These classifications were not part of routine clinical documentation and were not used during patient management.

### 2.3. Statistical Analysis

Descriptive statistics were used for analysis. Overall survival (OS) was calculated from the date of pathological diagnosis to death from any cause (for deceased individuals or until 4 July 2024, for survivors). The survival times of patients who were still alive at the time of the cutoff were right censored. Mortality data were sourced from the Institutional Cancer Center Registry and the Polish National Cancer Registry. Vital status was determined using the Institutional Cancer Registry and was cross-validated with the Polish National Cancer Registry. Patients with no recorded date of death in either registry as of 1 July 2024 were classified as alive.

No formal sample size calculation was performed, as this was a retrospective observational study including all eligible patients treated at the institution during the study period. The sample size was therefore determined by data availability rather than an a priori power calculation [12].

Survival distribution estimates were constructed via the Kaplan—Meier method, and differences in survival curves were evaluated via the log-rank test. Univariate analysis was used to assess the associations between OS and the following clinical risk factors at diagnosis: sex, age, number of metastatic sites, histopathology, prognostic risk groups (favorable vs. unfavorable), ECOG, LDH, ALP, Ca^2+^, CBC results, NLR, and PLR. Variables with minimal missing data relevant to initial patient presentation at diagnosis were selected. Univariate analysis identified predictors for OS. Factors with *p*-value < 0.10 were included in a multivariate model via the Cox proportional hazards model with backward stepwise selection. To avoid multicollinearity, composite variables combining ECOG performance status and LDH were not included in the multivariable Cox regression model. Variables of established clinical relevance were retained in the multivariable model regardless of borderline statistical significance.

Receiver operating characteristic (ROC) curves were used to identify cutoff points for the NLR and PLR. Although the results of the ROC analyses were not significant, the optimal cutoff values (determined by the Youden index) were 2.61 for the NLR and 174.79 for the PLR, which were chosen because of a lack of accepted thresholds. *p*-values and 95% confidence intervals were two-sided, with significance set at *p*-value < 0.05.

## 3. Results

### 3.1. Demographics and Clinicopathological Presentation

Among the 143 patients, the mean age was 59.02 ± 11.49 years (range 24–87), with a predominance of males (57.34%). Most patients (59.44%) presented with an ECOG performance status of 0–1 at treatment initiation. The primary metastatic sites identified were the lymph nodes (63.64%), liver (41.26%), bones (23.78%), lungs (21.68%), peritoneum (20.28%), subcutaneous tissue (15.28%), adrenal glands (9.09%), and other sites (21.68%).

The majority of pathological diagnoses were made by core biopsy (81 patients, 56.25%), and the rest had a cell block cytopathology (62 patients, 43.06%). This number changed significantly over time, as between 2006 and 2011, most of the pathology results were obtained only by fine needle aspiration (39 patients, 51.32%), and this number declined between 2012 and 2016 (23 patients, 34.33%) when a core biopsy/surgical biopsy became the gold standard according to international guidelines published in 2015 [11].

Patients with CUP were classified via postpathological examination as follows: adenocarcinoma (62 patients, 41.33%), squamous cell carcinoma (41 patients, 27.33%), poorly differentiated carcinoma (31 patients, 21.68%), and carcinomas with neuroendocrine differentiation (7 patients, 4.66%).

Fifty-three patients (38.46%) were categorized into specific clinicopathological subtypes [11]. This included 37 patients (25.87%) with squamous cell carcinoma in nonsupraclavicular cervical lymph nodes, 7 patients (4.90%) with solitary metastatic deposits from unknown primary, 5 patients (4.50%) with well-differentiated neuroendocrine carcinomas of unknown primary, 2 patients (1.40%) with poorly differentiated neuroendocrine carcinomas and 2 patients (1.40%) with a colorectal IHC (CK20 + CDX2 + CK7). Notably, 90 patients (62.94%) did not fit into any specific CUP subset. Further details are presented in Table 1.

### 3.2. Cancer Treatment

#### 3.2.1. Chemotherapy

Among the patients, 132 (92.31%) began first-line chemotherapy, with 41 receiving monotherapy and 91 receiving polychemotherapy (two or more drugs). Platinum-based regimens were the most prevalent (63.64%). Additional lines of chemotherapy were administered to a minority of patients: 46 patients (32.17%) received second-line treatment, and 9 patients (6%) received third-line treatment. Eleven patients were given only best supportive care. Those treated with platinum compounds exhibited the longest median survival of 8.8 months, compared with 4.83 months for other regimens and 1.3 months for best supportive care. Further details are summarized in Table 2.

#### 3.2.2. Radiotherapy

Nearly half of the patients (48.25%) underwent radiotherapy during their treatment. Most of these methods are palliative/symptomatic and target bone lesions (19.58%), central nervous system metastases (5.59%), or lymph nodes (5.59%). A smaller group received either sequential or concurrent radiotherapy alongside chemotherapy for potential “radicalization” (14.69%), primarily among patients with favorable subtypes of CUP. Further details are illustrated in Table 3.

#### 3.2.3. Survival Analysis

**Figure 1:**
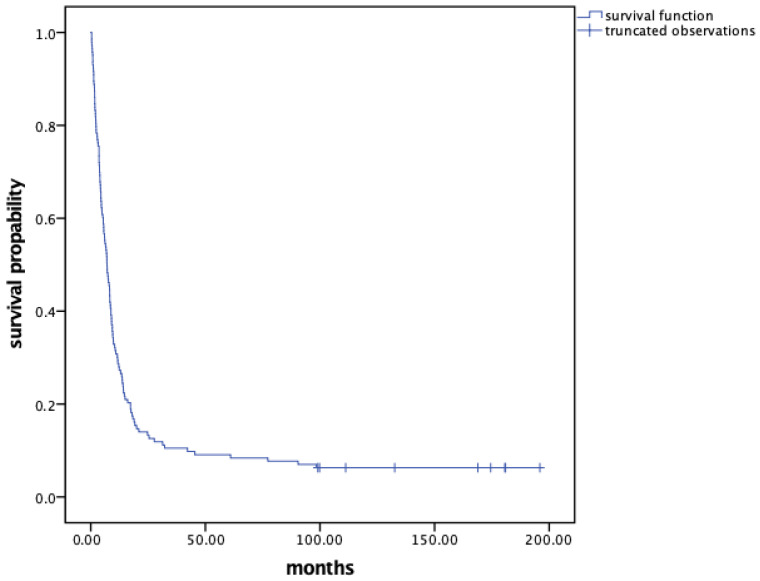
Kaplan—Meier survival curve for the study sample of 143 patients with CUP.

As of 1 July 2024, we observed 134 deaths (93.71%), with 9 patients being alive. The median overall survival for the cohort was 7.1 months [95% CI: 5.41–8.76], with a 3-year survival rate of 10.49%. The overall survival probability for the entire study sample is illustrated in Figure 1.

### 3.3. Univariate and Multivariate Analyses

Table 4 summarizes the median survival for different CUP patient subgroups. Univariate analysis revealed that multiple pretreatment clinical factors significantly influence patient survival.

Among the 14 preselected pretreatment variables, 12 were found to impact survival. Patients with fewer metastatic sites, undifferentiated or squamous cell carcinoma, an ECOG status of 0–1, normal LDH level (≤246 IU/L), favorable prognostic risk group, normal alkaline phosphatase level (≤120 IU/L), normal calcium level (≤2.65 mmol/L), normal hemoglobin level (>12 g/dL), normal platelet count (≤350 G/L), normal white blood cell count (<10 G/L), NLR < 2.61, and PLR < 174.79 had better outcomes.

In addition to the results from the univariate analysis, we utilized a multivariate Cox proportional hazards model with backward stepwise selection to identify factors independently associated with survival. This analysis revealed that ECOG score > 1 (HR = 1.69, 95% CI: 1.10–2.60, *p* = 0.016), elevated ALP (HR = 1.81, 95% CI: 1.20–2.74, *p* = 0.005), elevated calcium level (HR = 2.08, 95% CI: 1.05–4.13, *p* = 0.037), and NLR ≥ 2.61 (HR = 2.55, 95% CI: 1.67–3.89, *p* < 0.001) were independent predictors of poorer survival. Elevated LDH showed a consistent trend toward poorer survival but did not reach significance in the fully adjusted model (HR = 1.50, 95% CI: 0.99–2.27, *p* = 0.054). Other factors from univariate analysis (such as the number of metastatic sites did not significantly improve model fit when adjusting for other prognostic factors).

## 4. Discussion

Although cancer prognosis has improved in recent years, patients continue to seek clarity from their physicians regarding their treatment options and prognosis. Cancer of unknown primary (CUP) is considered an orphan disease [13] characterized by significant heterogeneity in clinical presentation, pathology, and mutational profiles. The diagnostic process for CUP is often lengthy, contributing to patient anxiety and potential delays in treatment.

Recent randomized trials incorporating comprehensive genomic profiling have emerged [14]; however, our current understanding of CUP largely relies on retrospective cohort studies and extrapolations. While the prevalence of CUP appears to be declining due to improved diagnostic algorithms, the median survival time in various Western countries ranges from 3 to 6 months [3,15], showing minimal improvement over time.

In light of this poor prognosis, we sought to analyze real-life data to identify pretreatment clinical factors that could influence survival among CUP patients.

Our cohort exhibited a median overall survival of 7.1 months, which aligns with findings from existing studies that reported survival times ranging from 3.5 to 16.5 months [16,17]. These variations can be attributed to differences in diagnostic practices, patient heterogeneity, and the limited evidence available for the timing of many previous studies. Epidemiological data frequently indicate poor prognoses, often not exceeding 6 months [15], whereas retrospective studies from academic institutions have recorded better outcomes, with some exceeding 1 year [18,19]. This discrepancy may arise from the fact that many newly diagnosed CUP patients succumb to the disease before reaching specialized treatment centers or presenting with poor performance status.

In our study cohort, the majority of patients were classified into the unfavorable prognostic risk group, mirroring the fact that up to 80% of CUP patients fall into this category [4]. These patients typically have a dismal prognosis, with most trials reporting modest benefits from platinum-based chemotherapy doublets [6,20,21,22].

In our population, a significant proportion (60.87%) of patients received platinum-containing chemotherapy, as this type of treatment remains the gold standard [4].

However, there was considerable variability in treatment regimens due to the time frame of our study (2006–2016), during which many treatment recommendations were not yet established, resulting in a more empirical approach. Although our institution does not have standardized protocols for second-line and subsequent treatments, 46 patients (32.17%) received second-line chemotherapy.

In the era of next-generation sequencing (NGS) and comprehensive genomic profiling (CGP), a deeper understanding of the mutational status of CUP allows for the identification of prognostic factors, such as CDKN2A deletion and RAS activation [18], as well as potential druggable targets [23,24].

Advances in molecular biology have fundamentally changed the clinical management of CUP, leading to the emergence of two distinct therapeutic concepts. Early applications of molecular diagnostics primarily focused on interfering the tissue of origin (TOO), with the intention of assigning patients to site-specific treatment regimens conventionally used for known primary malignancies. However, two randomized studies—GEFCAPI-04 and CUP-NGS—failed to demonstrate improvements in median progression-free survival (PFS) or OS when molecularly guided TOO-based therapy was compared with empiric chemotherapy [25,26].

More recently, attention has shifted toward CGP aimed at identifying actionable molecular alterations. This strategy enables the selection of targeted therapies or immunotherapies irrespective of the presumed primary site, representing a tumor-agnostic, site-independent approach.

Results from the CUPISCO trial evaluating molecularly guided treatment have also been reported; notably, this study randomized only patients who achieved stable disease or an objective response following first-line platinum-based chemotherapy (*n* = 438), and ultimately just 88 patients (20.1%) received matched targeted therapies [14].

Numerous trials exploring the role of immunotherapy in CUP have also shown promising results [9,10,27].

A systematic review and meta-analysis published in 2025 by Labaki et al., which included six studies—four of them randomized—demonstrated that the use of molecularly guided therapies was associated with improved OS in patients with CUP [28].

As the landscape of targeted treatments continues to evolve, more recent evidence has yielded mixed but encouraging results. The Chinese Fudan CUP-001 trial, unlike GEFCAPI-04 and CUP-NGS, reported a statistically significant prolongation of PFS in patients treated according to a TOO identified using a 90-gene expression assay [29]. Looking ahead, an integrated strategy combining molecular profiling for both tissue-of-origin determination and the detection of actionable alterations, followed by treatment allocation through a multidisciplinary tumor board (MTB), may represent a future direction. Supporting this concept, a French study reported a median OS of 18.6 months in patients receiving MTB-guided therapy compared with 11 months in those treated empirically (HR = 0.61, *p* = 0.04) [30].

Ongoing advances in molecular diagnostics, together with rapid developments in artificial intelligence and machine learning, have yielded increasingly promising results and are likely to reshape clinical approaches in the near future [31].

Owing to the gradual implementation of NGS and comprehensive genomic profiling for CUP patients at our institution since 2017, the analysis focused exclusively on cases diagnosed between 2006 and 2016. The introduction of novel diagnostic approaches and therapeutics, including immunotherapy, might influence overall patient survival and warrants future examination. In our retrospective cohort, no patients received immunotherapy or targeted therapies and molecular diagnostics tests were not used in the diagnostic evaluation of any patient. The analyzed cohort represents a pre-molecular profiling era, as comprehensive genomic testing and access to immunotherapy or targeted treatment for CUP were gradually implemented at our institution only after 2016.

During this study period (2006–2016), the ESMO guidelines regarding the CUP diagnostic process evolved. Recommendations emphasized the role of immunohistochemistry (IHC) in patients with poorly differentiated disease [32], including the addition of CK7 and CK 20 [33] or more robust IHC [34]. Although core biopsy provides enough material for robust pathological evaluation to become standard, our real-life evidence shows that CUP patient biopsies are often scarce or difficult to obtain due to technical reasons or tumor necrosis. On the basis of the aforementioned facts, many important CUP studies include patients diagnosed on the basis of cytopathological evaluation [7,35,36,37]. In our population, most patients were diagnosed with core biopsy with recommended more advanced IHC techniques over time.

We investigated clinical parameters widely available at the pretreatment stage for risk stratification. Our analysis revealed that poor performance status (ECOG 2–4), elevated alkaline phosphatase, elevated calcium, and elevated NLR were independently associated with poor survival in CUP patients, whereas elevated LDH showed a consistent trend toward poorer survival. Given its well-established prognostic relevance in CUP and other malignancies, LDH was retained in the multivariable model despite borderline statistical significance in the fully adjusted analysis.

Several studies have similarly attempted to identify various clinical parameters that impact disease progression. Factors such as leukocytosis [38], performance status [5,17,18,19,38,39,40], sex [18,19,41,42], the number of visceral organs involved [37,38,43], the number of organs affected [17,18,19,41,42], liver metastases [5,16,40], LDH levels [5], ALP levels [39], and the NLR [19,37,44] have all been shown to correlate with patient prognosis.

ALP elevation and hypercalcemia have been less studied in the context of CUP, and the NLR represents a more recent biomarker under investigation across multiple cancers [45].

Alkaline phosphatase is an enzyme that facilitates the hydrolysis of phosphate esters in alkaline environments. Elevated ALP levels have been correlated with tumor growth, metastasis, and invasion in various cancers [46,47].

In healthy individuals, the normal range for the neutrophil-lymphocyte ratio (NLR) is typically between 0.78 and 3.53 [48,49].

Previous studies have demonstrated the clinical relevance of the NLR as a biomarker in patients with unknown primary cancer; however, establishing a definitive cutoff point remains challenging.

As noted earlier, while the ROC models in our research did not show statistical significance, we found that the NLR value of 2.61 and PLR value of 174.79 effectively differentiated subjects in terms of survival outcomes.

Several approaches have been utilized to determine “high levels” of the NLR among cancer patients, with commonly referenced values ranging from 2 to 5 [19,37,44,50,51], whereas “high levels” of the PLR are typically reported between 150 and 300 [44,51,52,53]. This consistency with previous research supports the relevance of our findings.

However, further studies are necessary to validate the reliability of this parameter.

Hypercalcemia is a well-documented paraneoplastic syndrome associated with poor cancer prognosis [54]. Its elevation may also be related to the progression of bone metastases or a high tumor load. Furthermore, cancer treatment, which includes bisphosphonates, may influence patient prognosis [55]. Importantly, in our study cohort, only 12 patients presented elevated calcium levels; thus, these findings should be interpreted with caution.

In summary, to the best of our knowledge, this study represents the first analysis of CUP patients treated in Poland, and it includes one of the largest cohorts from Central/Eastern European countries receiving care at a specialized cancer center, with an assessment of important prognostic factors that may impact patient outcomes.

We also recognize the limitations of our study. As a retrospective cohort analysis, it is susceptible to selection and information biases. The retrospective nature of the analysis inherently limits control over data completeness and uniformity, as clinical and laboratory data were collected as part of routine clinical practice rather than a predefined research protocol. Although variables with minimal missing data were selected, residual information bias cannot be fully excluded. Repeated laboratory measurement and time-dependent covariates were not analyzed, as only baseline values obtained during diagnostic workup prior to treatment initiation were considered. Longitudinal analyses incorporating dynamic changes in parameters may provide additional insights.

Additionally, as this study was conducted at a single center and exclusively involved Polish patients, the results may not be generalizable to other geographic regions. Another variable that differs globally is the availability of treatments and the functioning of healthcare systems.

This study spans a lengthy period (a decade), during which diagnostic and treatment protocols evolved; however, a detailed temporal comparison was not feasible due to incomplete documentation for guideline adherence. The considerable variability in chemotherapy regimens may also affect the final outcomes observed in our research.

Furthermore, no molecular profiling or NGS data were available for this cohort. Although it allowed for the assessment of purely clinical prognostic factors, integration of clinical and molecular parameters represents an important direction for future research.

Finally, although overall survival was robustly ascertained using national registries, cause-specific mortality could not be evaluated. Future studies incorporating disease-specific outcomes or quality-of-life measures would provide a more valuable insight.

Future research should focus on prospective, multicenter studies to validate the prognostic value of routinely available clinical and laboratory parameters identified in this analysis. Integrating baseline clinical factors with molecular profiling and next-generation sequencing data may further refine risk stratification and patient selection.

## 5. Conclusions

In conclusion, our study offers valuable insights into patient survival, patient prognosis, and the clinical factors that affect individuals with cancer of unknown primary (CUP). This information can aid healthcare providers and patients during the initial evaluation for treatment eligibility. Additionally, these findings could support the establishment of regional or national CUP tumor boards, as recent research has indicated that such multidisciplinary teams can positively influence patient survival.

Moreover, this study provides a foundation for future analyses and treatment outcomes concerning molecular advancements in the diagnosis and treatment of patients with CUP.

## Figures and Tables

**Table 1:** Basic Demographic Data of the Study Sample.

Variable	*n* (%)
Total number of patients	*N* = 143
Mean age, years (range)	59.02 +/− 11.49 (24–87 years)
Gender	
Male	82 (57.34)
Female	61 (42.66)
Performance status	
ECOG 0–1	85 (59.44)
ECOG 2 or more	51 (35.66)
Metastatic sites	
Lymph nodes	91 (63.64)
Liver	59 (41.26)
Bones	34 (23.78)
Lungs	31 (21.68)
Peritoneum	29 (20.28)
Subcutaneous tissue	17 (11.89)
Adrenal Glands	13 (9.09)
Brain	8 (5.59)
Other	23 (16.08)
Number of metastatic sites	
1	52 (36.36)
2	47 (32.87)
≥3	44 (30.77)

ECOG—Eastern Cooperative Oncology Group.

**Table 2:** List of the Commonly Used First Line Chemotherapy Regimens among 132 patients with CUP.

**platinum compound (carboplatin, cisplatin, oxaliplatin)**	*N* = 84 (63.64%)
platinum compound monotherapy	18 (13.64%)
platinum compound plus	
etoposide	14 (10.61%)
fluorouracil	20 (15.15%)
doxorubicin + cyclophosphamide	8 (6.06%)
paclitaxel	8 (6.06%)
gemcitabine	5 (3.79%)
vinorelbine	2 (1.52%)
other	9 (6.82%)
**other polychemotherapy regimens**	*N* = 25 (18.94%)
FAM	11 (8.33%)
FAC	5 (3.79%)
CAV	3 (2.27%)
other	6 (4.55%)
**monotherapy**	*N* = 23 (17.42%)
gemcitabine	8 (6.06%)
fluorouracil	6 (4.55%)
methotrexate	3 (2.27%)
paclitaxel	3 (2.27%)
other	3 (2.27%)

FAM (doxorubicin, fluorouracil, mitomycin), CAV (vincristine, cyclophophamide, doxorubicin), FAC (fluorouracyl, doxorubicin, cyclphosphamide).

**Table 3:** Types of Radiotherapy Used Among Patients with CUP.

Radiotherapy		*N* = 69	48.25%
Palliative			
	bones	28	19.58%
	CNS	8	5.59%
	lymph nodes	8	5.59%
	VCSS	1	0.70%
	other	3	2.10%
“Definitive”			
	sequential or concurrent with chemotherapy	18	12.59%
	adjuvant after surgical excision	3	2.10%

CNS—central nervous system, VCSS—vena cava superior syndrome.

**Table 4:** Univariate and Multivariate Analyses of Overall Survival.

Variable	*n*	Median Survival (Months)	3-y Survival (%)	Univariate Analysis	Multivariate Analysis	
				*p*-Value	*HR*	*p*-Value
**sex**				0.268		
female	61	8.2	9.84			
male	82	5.73	8.54			
**age**				0.441		
<65	101	7.13	11.88			
≥65	42	7.03	7.14			
**metastatic organs**						
1	52	9.1	21.15			
2	47	7.17	2.13	0.007		
≥3	44	3.97	6.82	0.001		
**histopatology subtype**						
undifferentiated	31	12.2	9.68			
squamous cell	41	8.8	24.39	0.811		
neuroendocrine	7	5.23	0	0.165		
adenocarcinoma	62	4.3	3.23	<0.001		
**prognostic risk group (ECOG + LDH)**				<0.001		
favorable	55	10.63	21.82			
unfavorable	77	4.47	2.6			
**LDH**				<0.001	1.50	0.054
normal	73	9.03	16.44			
elevated	57	4.83	3.51			
**ECOG**				<0.001	1.69	0.016
0–1	85	9.4	15.29			
≥2	51	3.6	1.96			
**ALP**				<0.001	1.81	0.005
normal	73	9.6	15.07			
elevated	62	4.3	4.84			
**calcium level**				0.001	2.078	0.037
normal	122	7.17	11.48			
elevated	12	3.17	0.0			
**Hb level (g/dL)**				0.032		
normal	99	8.53	12.12			
<12	44	5.67	6.82			
**PLT**				0.037		
normal	97	8.23	13.4			
>350 (ULN)	46	4.93	4.35			
**WBC**				0.03		
normal	74	8.8	13.51			
>10 (ULN)	69	4.63	7.25			
**NLR**				<0.001	2.550	<0.001
<2.61	49	13.7	24.49			
≥2.61	85	4.4	2.35			
**PLR**				0.003		
<174.79	72	8.8	16.67			
≥174.79	62	5.73	3.23			

LDH—serum lactate dehydrogenase, ALP—serum alkaline phosphatase, Hb—hemoglobin, PLT—platelet count, WBC—white blood count, NLR—neutrocyte to lymphocyte ratio, PLR—platelet to lymphocytes ratio, ECOG—Eastern Cooperative Oncology Group performance status.

## Data Availability

The data presented in this study are retrospective clinical data and are not publicly available due to ethical restrictions, patient confidentiality, and data protection regulations. The data may be made available from the corresponding author upon reasonable request and with appropriate institutional approval.

## Abbreviations

The following abbreviations are used in this manuscript:

CUP	Cancer of unknown primary
LDH	Lactate dehydrogenase
ECOG	Eastern cooperative oncology group performance status
NGS	Next-generation sequencing
RWE	Real-world evidence
CBC	Complete blood count
Ca^2+^	Blood calcium level
LDH	Blood lactate dehydrogenase
ALP	Alkaline phosphatase
NLR	Neutrophil-lymphocyte ratio
PLR	Platelet-lymphocyte ratio
OS	Overall survival
HR	Hazard ratio
CI	Confidence interval
ROC	Receiver operating characteristic
*p*-value	Probability value
CGP	Comprehensive genomic profiling
TOO	Tissue of origin
PFS	Progression-free survival
